# The Morphometric Study of Degenerative Lateral Canal Stenosis at L4–L5 and L5–S1 Using Magnetic Resonance Imaging (MRI): Feasibility Analysis for Posterior Surgical Decompression

**DOI:** 10.5704/MOJ.1503.015

**Published:** 2015-03

**Authors:** MI Yusof, MSM Shif, MS Abdullah

**Affiliations:** Department of Orthopaedics, Universiti Sains Malaysia, Kubang Kerian, Malaysia; * Department of Radiology, Universiti Sains, Malaysia, Kubang Kerian, Malaysia

## Abstract

This study was to evaluate the morphological features of degenerative spinal stenosis and adequacy of lateral canal stenosis decompression via unilateral and bilateral laminectomy. Measurements of facet joint angulation (FJA), mid facet point (MFP), mid facet point distance (MFPD), the narrowest point of the lateral spinal canal (NPLC) and the narrowest point of the lateral spinal canal distance (NPLCD) were performed. At L4L5 of the right and left side, the mean distance between the lateral border of the dura and MFP was 1.0 ± 0.2 cm and 1.0 ± 0.3cm respectively. The mean NPLC was seen at 0.7 ± 0.3 and 0.7 ± 0.3 cm cm from the dura. At L5S1 of the right and left side, the mean distance between the lateral border of the dura and MFP was 1.2± 0.2 and 1.3 ± 0.2 cm respectively. The mean NPLC was seen at 0.8 ± 0.4 and 0.9 ± 0.5 cm from the dura. Unilateral laminectomy may result in incomplete decompression.

## Introduction

Degenerative lumbar disease with lateral canal stenosis among elderly patients is not uncommon and most commonly involved the L4-L5 and L5-S1 levels^1–3^. It represents different stages of degenerated spinal disease with the involvement of intervertebral discs, vertebra bodies, ligamentum flavum, posterior longitudinal ligament, facet joints and the spinal venous plexus^3–6^.

Presence of lateral stenosis is often missed or underestimated. Lateral canal stenosis causes compression on the nerve root that passes through the affected intervertebral foramen. Relatively minimal degree of stenosis may cause significant stenotic symptoms if it involves the lateral canal but can be less symptomatic if the stenosis is only confined to the central spinal canal^3^. Failure to address the lateral stenosis component of the patients’ symptoms had contributed to the increased rate of poor surgical outcomes associated with incomplete decompression. The contributing factors for these problems include lack of understanding of the pathophysiology, its importance in the management and inaccurate reported MRI findings^7–8^.

Adequate decompression and preservation of spinal stability are the two main prerequisites for successful neural decompression. The exact location of the nerve root compression must be confirmed before decompression. Hypertrophic facet joint is an important element of lateral spinal stenosis. Adequate facet joint resection is therefore necessary to decompress the lateral canal^9^. It is crucial that in the enthuthiasm of performing surgical decompression, 50% of the facet joint is preserved to avoid spinal instability^9–11^ The concern of the feasibility for lateral canal decompression arises based on the reports of high incidence of failed back syndrome due to failure to address lateral foraminal stenosis using ipsilateral laminectomy^9–12^ Even, with bilateral laminectomies performed, the incidence of inadequate lateral stenosis decompression was relatively high leading to persistence of neurological symptoms post operatively^13^.

It is imperative to understand the pathoanatomy of lateral spinal stenosis and appropriate surgical approach for decompression^14,15^ We believe that the surgical approach to the lateral canal provided by laminectomy is significantly determined by the exact location of the nerve root compression^9,16^, severity of compression and preservation of spinal stability, which are substantially contributed by the facet joints. Adequate knowledge on the pathoanatomy of the lateral canal will assist the surgeons to understand the rationale of the surgical approach and achieve adequate nerve decompression without causing spinal instability^3^ Based on these reasons, we would like to investigate the morphological features of degenerative lateral spinal stenosis and the feasibility of surgical decompression from posterior approach.

## Materials and Methods

The aim of this study is to study the morphology of degenerative lateral spinal stenosis. The specific objectives of this study are to i) locate the narrowest part of the lateral canal in this population ii) examine the association between the location of stenosis and lumbar facet joint angulation and iii) assess the feasibility of lateral canal posterior decompression via unilateral and bilateral laminectomy.

This morphological study involved measurement of relevant parameters that had been identified to achieve the study objectives. The patients who were enrolled in this study include all patients who had been confirmed to have degenerative spinal stenosis based on clinical and radiological evaluation. Their data were extracted from our spine clinic registry from 2008-2010. Those who had evidence of having other than degenerative lumbar stenosis were excluded from this study.

**Fig. 1:**
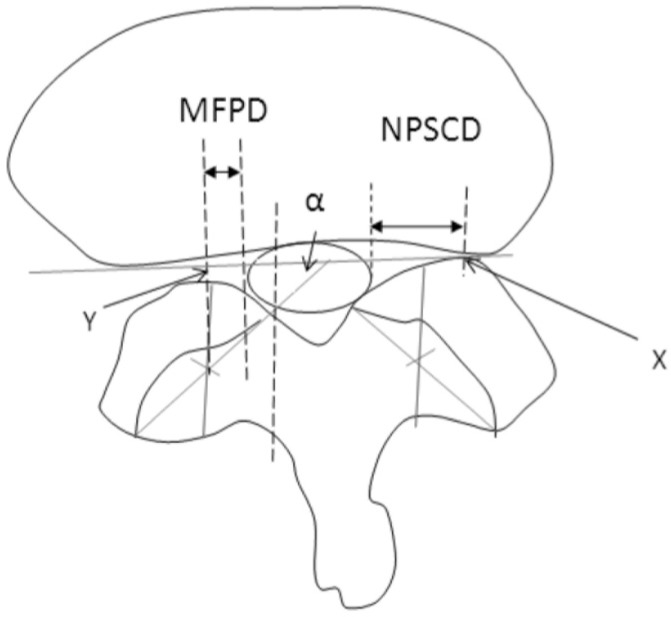
Measurement of the parameters involved; α, Facet Joint Angle (FJA), X, Narrowest Point of Spinal Canal (NPSC), Y, Mid Facet Point (MFP), Narrowest Point of Spinal Canal Distance (NPSCD) and Mid Facet Point Distance (MFPD).

Computerized measurements were performed on their axial magnetic resonance images (MRI) (GE Medical Systems, Milwaukee,WI) at the level of the lateral canal. The parameters measured were i) facet joint angulation (FJA), ii) mid facet point (MFP), iii) mid facet point distance (MFPD), iv) the narrowest point of the lateral spinal canal (NPLC) and the narrowest point of the lateral spinal canal distance (NPLCD). (Figure 1)

FJA was defined as the angle between the facet articular surface and the horizontal line perpendicular to the spinous process. MFP was determined by dividing the facet articular into two halves perpendicularly to the coronal plane. NPLC was determined by the point at the facet joint that forms the narrowest part of the lateral spinal canal. The MFPD and NPLCD were obtained from the measurements of these two points to the lateral border of the dura.

The location of NPLC was then divided into three types; medial to MFP, lateral to MFP or at the MFP. Feasibility of complete decompression is defined as accessibility of this point from the ‘laminectomy window’ with preservation of 50% (lateral half) of the ipsilateral facet joint.

The measurements were made at L4L5 and L5S1 levels bilaterally. The statistical analysis was performed using SPSS software (version 20; SPSS, Inc., Chicago, IL).

## Results

There were 80 patients enrolled in this study, with 320 facets and lateral canals scrutinized. There were 47 males and 33 female patients involved with their average age were 58.6± 9.9 years old.

The mean measurements of all parameters were summarized in (Table I).

L4-L5 Level : At L4-L5 level, there were 74/80 (93%) stenotic lateral canals on the right and on the left sides.

On the right side, the mean distance between the lateral border of the dura and MFP was 1.0 ± 0.2 cm. The mean NPLC was seen at 0.7 ± 0.3 cm from the dura. The prevalence of NPLC medial, lateral and at the MFP were 63/74, 9/74 and 2/74 respectively.(Table II)

On the left side, the mean distance between the lateral border of the dura and MFP was 1.0 ± 0.3cm. The mean NPLC was seen at 0.7 ± 0.3 cm from the dura. The prevalence of NPLC medial, lateral and at MFP were 62/74, 9/74 and 3/74 respectively.

The mean facet joint angles were 45.4 ± 9.2 and 44.4 ±8.4 degrees at the right and left sides respectively.

L5-S1 level: At L5S1 level, there were 34/80 (43%) stenotic lateral canals on the right and on the left sides.

On the right side, the mean distance between the lateral border of the dura and MFP was 1.2± 0.2 cm. The mean NPLC was seen at 0.8 ± 0.4 cm from the dura. The prevalence of NPLC medial, lateral and at the MFP was 27/74, 6/74, 1/74 respectively.

On the left side, the mean distance between the lateral border of the dura and MFP was 1.3 ± 0.2 cm. The mean NPLC was seen at 0.9 ± 0.5 cm from the dura. The prevalence of NPLC medial, lateral and at MFP were 28/74, 4/74 and 2/74 respectively.

The mean facet joint angles were 42.9± 7.5 and 42.6 ± 6.5 degrees at the right and left sides respectively. There was no significant difference between the L4L5 and L5S1 levels in term of the mean of MFPD, NPLCD and FJA bilaterally.

Statistical analysis did not show any significant correlation between the NPLC and FJA at L4-L5 and L5-S1 bilaterally. This finding indicated that the location of stenosis was not influenced by the facet joint angle.

## Discussion

Central canal stenosis is commonly associated with lateral stenosis and has been well described^14,17^ Anatomically, the lateral canal can be divided into three zones; the entrance (lateral recess), mid (foraminal) and exit (extraforaminal) zones.^15^. The surgeons must understand the anatomical abnormalities of the lateral canal because failure to do so would lead to inaccurate diagnosis and incomplete spinal decompression.

**Table I:** The mean mid facet point (MFP), narrowest point of lateral canal (NPLC) and facet joint angle (FJA) at L4–L5 and L5–S1 levels

MFP distance(cm)				NPLC distance (cm)				FJA (deg)			
	L4–L5		L5–S1		L4–L5		L5–S1		L4–L5		L5–S1	
	R	L	R	L	R	L	R	L	R	L	R	L
mean	1.0	1.0	1.2	1.3	0.7	0.7	0.8	0.9	45.4	44.4	42.9	42.6
SD	0.2	0.3	0.2	0.2	0.3	0.3	0.4	0.5	9.2	8.4	7.5	6.5

**Table II:** The location of narrowest point of lateral canal (NPLC) in relation with the location of mid facet point (MFP) at L4–L5 and L5–S1

Level	Narrowest point of lateral canal (NPLC)					
	Medial		Midfacet		Lateral	
	R	L	R	L	R	L
L4–L5	63/74	62/74	2/74	3/74	9/74	9/74
L5–S1	27/34	28/34	1/34	2/34	6/34	4/34

**Table III:** The distance between narrowest point of lateral canal and mid facet point (NPLC– MFP distance) at L4–L5 and L5–S1 levels

	NPLC– MFP distance (cm)			
	L4–L5		L5–S1	
	R	L	R	L
Mean	0.4	0.4	0.5	0.5
SD	0.2	0.2	0.3	0.3

Lateral stenosis is common among our patients undergoing surgical decompression. Posterior decompression with laminectomy alone, either performed using traditional technique or mimimally invasive technique, will not give satisfactory results if the presence of lateral stenosis (foraminal and extraforaminal) is not addressed adequately. NPLC is the area where the nerve root is most severely compressed and primarily needs decompression. Inability to address this area during the surgery will result in incomplete neural decompression and poor outcomes.

Based on our findings, most of our patients had lateral canal stenosis involving both sides of the L4–L5 and L5–S1 levels. These findings indicated that in degenerative spinal stenosis, the lesions are not confined to one side and more often involved bilaterally, which were reflected from their clinical findings. Thus, bilateral decompression is required in most of the patients with lateral canal stenosis. Since L4–L5 level is commonly affected, it is paramount that L5–S1 involvement is ruled out in all the patients with degenerative stenosis.

The average distances between MFP from the dura were 1.0 and 1.2 cm at L4‐L5 and L5‐S1 respectively. Therefore, the surgeons must not extend facetectomy beyond this distance to avoid significant facet damage and spinal instability. Since, the average location of NPLC was 0.7‐ 0.9 cm (medial to MFP), surgical decompression is technically possible in most of the patients without causing spinal instability. However, 23/296 (7.8%) NPLC at L4L5 and 13/136 (9.6%) at L5S1 were located at or lateral to the MFP. Therefore in this group of patients, careful decompression of the nerve roots is required because resection of the hypertrophic facet needs to be extended more laterally to achieve satisfactory decompression. This would put the facet joints more vulnerable to significant damage and potential spinal instability. Spinal instability may not be affected if NPLC is located more medially to the MFP which was seen in the majority of patients in our studied population.

**Fig. 2:**
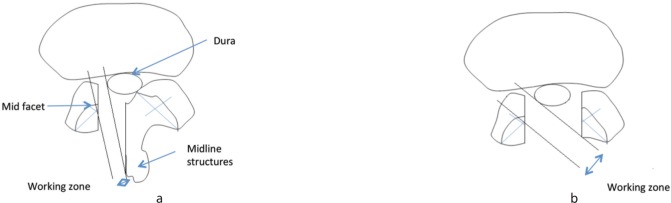
Anatomical restrictions for lateral canal decompression (a) ipsilaterally (unilateral laminectomy) and (b) contralaterally (bilateral laminectomy); the dura anteriorly, mid facet joint (MFP) laterally and mid line structures (spinous process, interspinous and supraspinous muscles) posteriorly. Working zone is an area between the lateral border of the dura and MFP. a b Feasibility Analysis for Posterior Surgical Decompression.

NPLC-MFP distance measurement which ranged between 0.4–0.5 cm provided a parameter which indicated the proximity of the most stenotic area and the middle of facet joint. (Table III)Therefore, resection of the facet joint 0.5 cm lateral from the compressed nerve would result in spinal instability. However, if NPLC- MFP distance was very small, more than 50% of facet joint would be inadvertently damaged in the process of decompression of the medially located NPLC. If we presume that NPLC- MFP distance of 0.2 cm and less is significantly near, our findings showed that at L4–L5 and L5–S1, 23/125 (18%) and 5/55 (9%) of the patients in this group were at risk of developing spinal instability following lateral canal decompression surgery. Therefore, the surgeons should choose an appropriate approach and technique to remove the facet adequately without sacrificing spinal stability. (Figure 2)

**Fig. 3:**
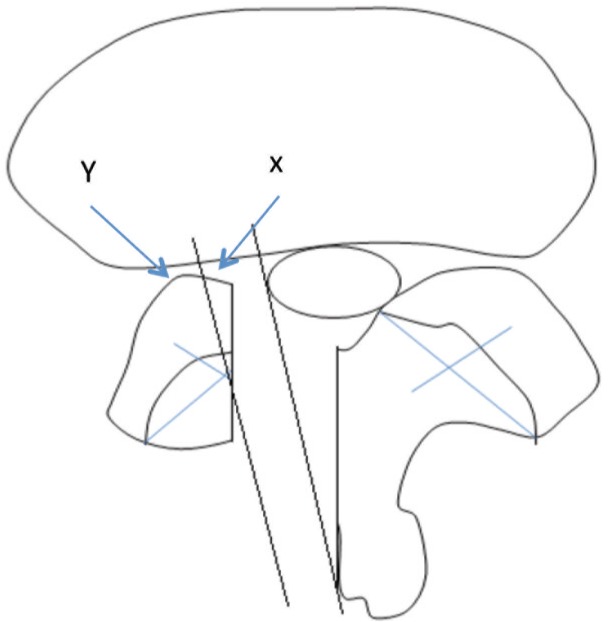
Ipsilateral laminectomy: If the NPLC is located medially (x) to the MFP, complete decompression can be done with minimal lateral angulation. However, if the NPLC is located at or more laterally (Y), NPLC will not be addressed adequately because more than half of the facet must be removed to reach this point.

**Fig. 4:**
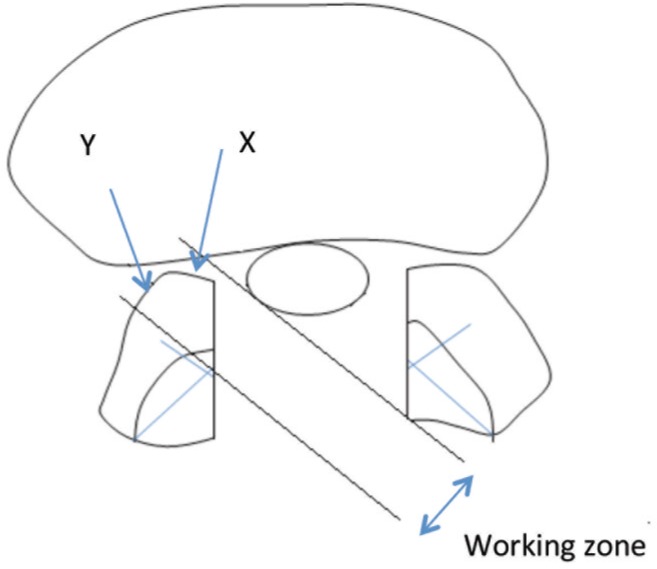
Contralateral laminectomy: NPLC which are located medially, at or more laterally to MFP can be addressed adequately using this approach as more than half of the medial facet can be preserved with complete resection of NPLC. Note that, this approach provides wider working zone for safer and efficient decompression.

Complete canal decompression may be achieved if the NPLC is located medially to the MFP with minimal lateral angulation via ipsilateral laminectomy. NPLC which is located at or more laterally to the MFP cannot be addressed adequately because more than 50% of the medial facet must be removed to reach this point. (Figure 3) Working zone is also narrow with this approach. Lateral canal decompression using ipsilateral approach is therefore difficult or impossible. In this condition, lateral stenosis decompression is best performed from the contralateral side since it only involves undercutting the anteromedial part of the superior articular process of the facet joint. More than 50% of the facet joint can be preserved more effectively by this approach and therefore spinal stability can be maintained. Bilateral laminectomy allows decompression of lateral stenosis both sides performed from the contralateral side. (Figure 4)

**Fig. 5:**
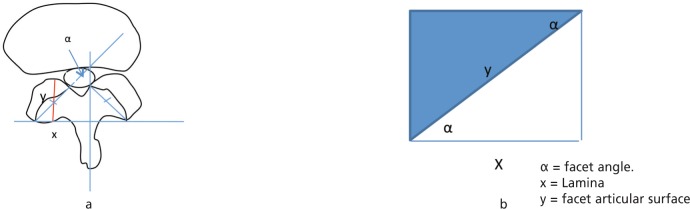
Estimation of facet joint resection via vertically performed laminectomy. Fig. a is mathematically represented by Fig. b. Facet articular surface cutting = amount of laminectomy/ cos facet angle. Since average facet angle is 42 degrees, 1 mm laminectomy done vertically via ipsilateral laminectomy causes 1.4 mm facet joint resection.

Amount of facet damage can be estimated using a mathematical calculation. (Figure 5) Facet joint articular surface resection is directly proportionate to the amount of laminectomy performed, depending on the facet joint angle. Generally, vertically performed laminectomy would cause more damage to the facet joint than laminectomy performed from the contralateral side. Using mathematical calculation, the amount of facetectomy resulted from 1 mm laminectomy can be estimated. Every 1 mm laminectomy done vertically (e.g. via ipsilateral laminectomy) results in 1.4 mm facet joint resection based on the calculation. (Figure 5) On the other hand, the amount of facet joint damage is less when performed from the contralateral side. The working zone is also wider which makes the procedure easier and safer. High incidence of dural tear and neurological symptoms associated with ipsilateral approach could be due to narrow working zone provided by the approach, needing excessive dura or nerve root retraction during decompression^18–20^.

**Fig. 6:**
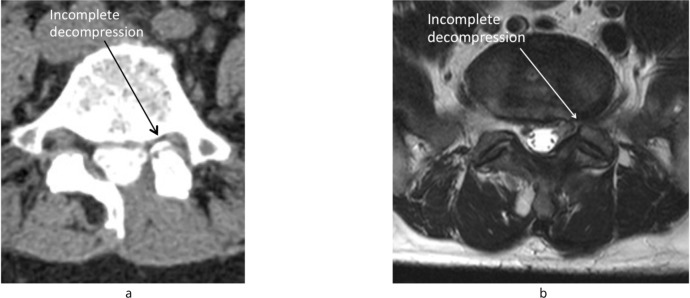
These post-operative images of patients underwent decompression for degenerative spine stenosis using unilateral laminectomy. Difficulty to decompress ipsilateral lateral canal stenosis at L4–L5 (a) and L5–S1 (b) levels caused incomplete decompression and residual symptoms.

Unilateral laminectomy with ipsilateral decompression is reported to be effective for central canal or lateral recess stenosis^21–24^ However, the adequacy of decompression is questionable for true lateral stenosis. The main limitation of this technique is the difficulty of achieving complete decompression, especially for far lateral stenosis. Removal of the osteophytes or hypertrophied facet at these areas may damage the facet joints and cause spinal instability. Based on this analysis, bilateral lateral stenosis using unilateral laminotomy approach may leave the patient with residual symptoms on the uncompressed side, especially of the ipsilateral side. Due to its minimal exposure, this technique may result in incomplete decompression and higher complications in approximately 8‐10% of patients. An additional 9‐18% of patients are potentially at risk due to proximity of mid facet and point of stenosis. Contralateral approach is more reasonable as it allows ‘undercutting’ the hypertrophied facet. The amount of facet removal is more predictable and therefore adequate decompression can be achieved and spinal stability can be preserved.

Our study may not be very accurate as there are many limitations associated with the number of samples and imaging technique. Future studies are necessary to determine if these measurements actually do correlate with clinical outcomes in patients with symptomatic lumbar spinal stenosis.

## Conclusion

Complete decompression and preservation of spinal stability are both important for improved functional outcomes postoperatively. Due to its minimal exposure, this technique may result in incomplete decompression and higher complications in approximately 8–10% of patients. An additional 9–18% of patients are potentially at risk due to proximity of mid facet and point of stenosis. Contralateral approach is more reasonable as it allows ‘undercutting’ the hypertrophied facet. The amount of facet removal is more predictable and therefore adequate decompression can be achieved and spinal stability can be preserved.
